# Potential roles for mitochondria-to-HSF1 signaling in health and disease

**DOI:** 10.3389/fmolb.2023.1332658

**Published:** 2023-12-18

**Authors:** Johnathan Labbadia

**Affiliations:** Department of Genetics, Evolution and Environment, Division of Biosciences, Institute of Healthy Ageing, University College London, London, United Kingdom

**Keywords:** HSF1, protein homeostasis, mitochondria, ageing, development, disease

## Abstract

The ability to respond rapidly and efficiently to protein misfolding is crucial for development, reproduction and long-term health. Cells respond to imbalances in cytosolic/nuclear protein homeostasis through the Heat Shock Response, a tightly regulated transcriptional program that enhances protein homeostasis capacity by increasing levels of protein quality control factors. The Heat Shock Response is driven by Heat Shock Factor 1, which is rapidly activated by the appearance of misfolded proteins and drives the expression of genes encoding molecular chaperones and protein degradation factors, thereby restoring proteome integrity. HSF1 is critical for organismal health, and this has largely been attributed to the preservation of cytosolic and nuclear protein homeostasis. However, evidence is now emerging that HSF1 is also a key mediator of mitochondrial function, raising the possibility that many of the health benefits conferred by HSF1 may be due to the maintenance of mitochondrial homeostasis. In this review, I will discuss our current understanding of the interplay between HSF1 and mitochondria and consider how mitochondria-to-HSF1 signaling may influence health and disease susceptibility.

## Introduction

Maintaining protein homeostasis (proteostasis) (i.e., an environment free of misfolded, mislocalised and aggregated proteins) is critical for cells to function properly (Labbadia and Morimoto, 2015). This is achieved through the action of the Proteostasis Network (PN), a dynamic collection of protein quality control factors that monitor and correct any errors in protein synthesis, folding or localisation that arise across the cell. Throughout life, demands on the PN can fluctuate due to increased growth and protein synthesis, changes in nutrient availability, the presence of genomic mutations, or altered environmental conditions (e.g., increased temperature, reduced pH, etc.). This can overwhelm the PN and lead to the appearance of misfolded, mislocalised and aggregated proteins within cells (often collectively referred to as proteostasis collapse) (Labbadia and Morimoto, 2015). When this persists, cells can malfunction and die, leading to developmental lethality, reproductive defects or tissue dysfunction and disease (Hipp et al., 2019).

To protect against this, cells have evolved highly conserved stress response pathways that rapidly augment the PN through the activation of specific transcriptional programs. Among these, the Heat Shock Response (HSR) is induced in response to the accumulation of misfolded proteins in the cytosol/nucleus by the transcription factor, Heat Shock Factor 1 (HSF1) (Gomez-Pastor et al., 2018). HSF1 activity is controlled through interactions with molecular chaperones, structural changes in HSF1, post-translational modifications and chromatin state, thereby allowing for rapid and precise transactivation at genes encoding molecular chaperones, proteasomal subunits and other PN components, followed by rapid transcriptional attenuation once proteostasis is restored (Vihervaara et al., 2018; Kmiecik and Mayer, 2022). This process is critical for the maintenance of cell viability under conditions of protein folding stress. In addition, HSF1 has been shown to drive a basal transcriptional program that is distinct from the HSR and crucial for development and reproduction (Li et al., 2017). Consistent with these roles, increased HSF1 activity promotes longevity and suppresses the appearance and progression of several age-associated protein conformational diseases (Gomez-Pastor et al., 2018). However, the relationship between HSF1 activity and organismal health is complex, with increased HSF1 activity also associated with cancer cell survival and tumorigenesis (Dai and Sampson, 2016).

Given that the most prominent hallmark of HSF1 activity is an increase in levels of cytosolic molecular chaperones, such as HSP70, small heat shock proteins (sHSPs), HSP40, TRiC subunits and HSP90, it is reasonable to assume that HSF1 influences long-term organismal health by protecting the cytosol/nucleus against protein misfolding. However, recent evidence shows that HSF1 activity is also crucial for maintaining mitochondrial function through the activation of cytosolic and mitochondrial unfolded protein responses that are distinct from the HSR (Song et al., 2021). This suggests that at least some of the beneficial effects stemming from increased HSF1 activity may arise from preserving or restoring mitochondrial homeostasis.

In this mini-review, I will summarize our current understanding of the interplay between HSF1 and mitochondria, and discuss what these mechanisms mean for our understanding of different diseases and our ability to promote human health. Excellent reviews providing in-depth explanations of the mechanisms by which the PN operates, the regulation of HSF1, and the relationship between proteostasis, development, ageing and disease, can be found elsewhere (Li et al., 2017; Gomez-Pastor et al., 2018; Vihervaara et al., 2018; Kmiecik and Mayer, 2022).

### HSF1 activity and mitochondrial homeostasis are intimately coupled

Over 99% of mitochondrial proteins are encoded by nuclear genes and are post-translationally imported into mitochondria as preproteins (Pfanner et al., 2019). As such, cytosolic chaperones, such as HSP70 and HSP90 (the levels of which are regulated by HSF1), are central to this process, by engaging with mitochondrially targeted peptides at the ribosome and chaperoning them to dedicated import machineries at the mitochondrial outer membrane (Song et al., 2021).

Defects in mitochondrial import lead to an accumulation of preproteins in the cytosol. These are highly aggregation prone and form cytosolic deposits that disrupt proteostasis and further compromise mitochondrial function (Nowicka et al., 2021). To combat this, yeast can enhance cytosolic proteostasis capacity through elevated proteasomal activity and an HSF1-dependent increase in levels of molecular chaperones (Wang and Chen, 2015; Wrobel et al., 2015; Boos et al., 2019), thereby guarding against the toxic effects of orphaned preproteins, while attempting to restore import. Similarly, impairment of the mitochondrial electron transport chain (ETC), knockdown of mitochondrial HSP70 (mtHSP70) or loss of the mitochondrial outer membrane insertase, MTCH-1, are associated with increased levels of cytosolic sHSPs and HSP70 family members in *C. elegans* and *Drosophila* (Kim et al., 2016; Borch Jensen et al., 2017; Williams et al., 2020; Aman et al., 2022).

In addition to elevating levels of cytosolic chaperones, HSF1 has also been found to promote the mitochondrial unfolded protein response (UPR^mt^) in worms and human cells subjected to mitochondrial stress. When mtHSP70 is knocked down in *C. elegans*, HSF-1 (the name of the sole heat shock factor in *C. elegans*) cooperates with the transcription factors DVE-1/SATB1 to drive the expression of UPR^mt^ genes (Kim et al., 2016). In addition, treatment of mouse embryonic fibroblasts (MEFs) with GTPP (an inhibitor of the matrix HSP90/TRAP1) or CDDO (an inhibitor of the matrix LON protease) results in the up-regulation of HSP60 and mtHSP70 in an HSF1 dependent manner (Katiyar et al., 2020; Sutandy et al., 2023).

Finally, HSF1 also maintains NAD+ (a metabolite that is crucial for increased mitochondrial biogenesis) levels in the livers of fed and fasted mice by directly binding to, and increasing the expression of, the key NAD+ salvage pathway enzyme, nicotinamide phosphoribosyltransferase (NAMPT) (Qiao et al., 2017). Conversely, increased PGC1a activity in fasted mouse livers directly represses the expression of HSF1 target chaperone genes (Minsky and Roeder, 2015). This suggests an interesting interplay between mitochondrial states, where stress and biogenesis have opposing effects on HSF1 activity.

Together, these observations make it clear that HSF1 is important not just for cytosolic proteostasis, but also for efficient mitochondrial biogenesis and maintenance. Consistent with this, mutations in HSF cause mitochondrial import defects in yeast (Smith and Yaffe, 1991) and the loss of HSF1 results in mitochondrial dysfunction, REDOX imbalance and impaired mitochondrial biogenesis in mice (Yan et al., 2002; Bierkamp et al., 2010; Qiao et al., 2017).

### Mitochondria increase HSF1 activity through diverse mechanisms

**FIGURE 1:**
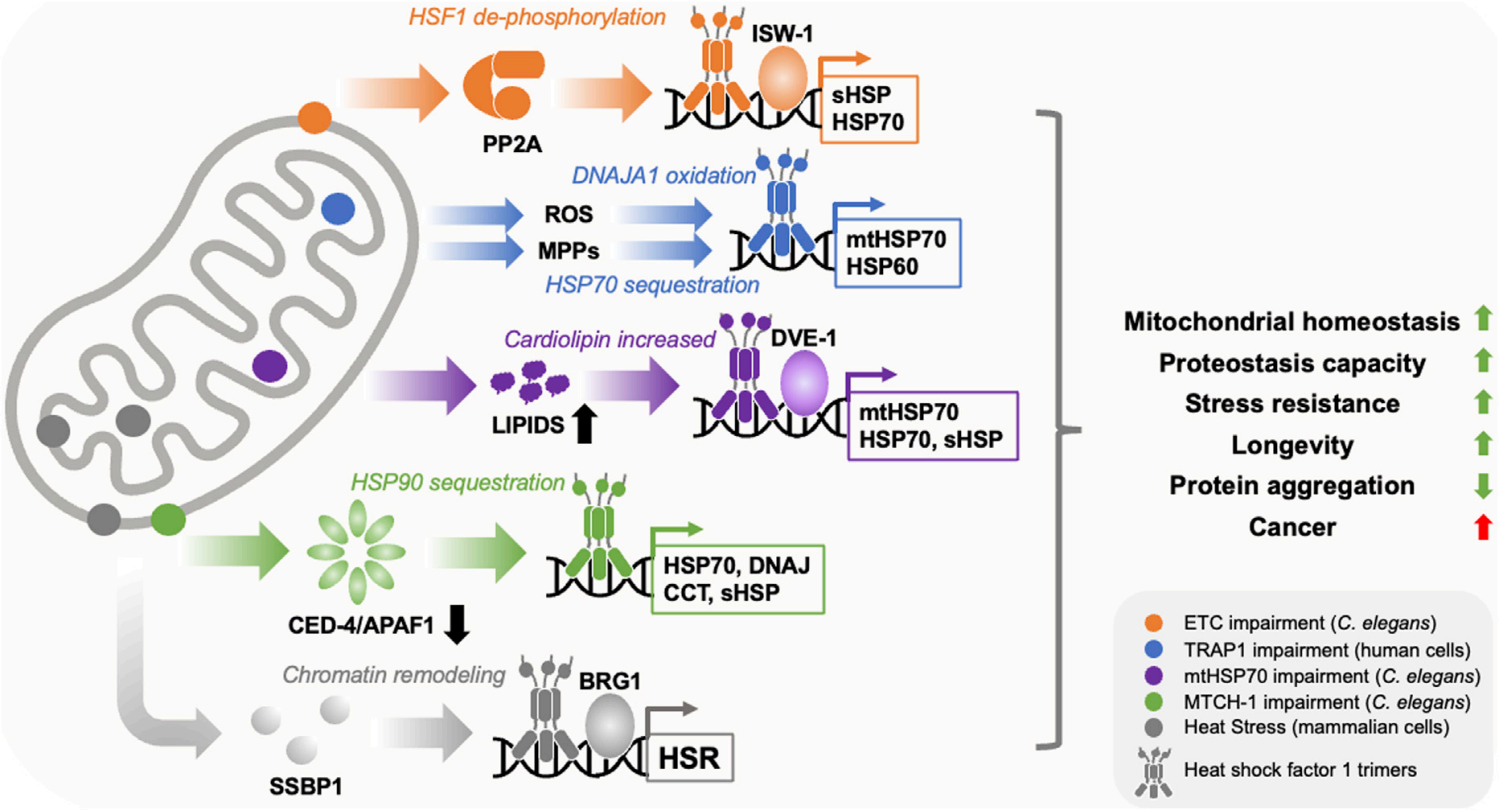
Mitochondria regulate HSF1 activity through diverse mechanisms. Mitochondria utilise diverse mechanisms to regulate HSF1 activity in response to diffferent types of stress (depicted by different colours and locations within the cartoon mitochondrion). These include the translocation of mitochondrial single strand binding protein (SSBP1) to the nucleus, dephosphorylation of HSF1 by Protein Phosphatase 2A (PP2A), sequestration of molecular chaperones such as HSP70 and HSP90, depletion of the programmed cell death factor CED-4/APAF1, activation of the transcription factor DVE-1, release of reactive oxygen species (ROS) and mitochondrial precursor protein (MPP) accumulation in the cytosol, and chromatin remodelling through ISW-1 and BRG1. This produces overlapping but distinct transcriptional responses that enhance cytosolic and mitochondrial proteostasis capacity, restore mitochondrial homeostasis, reduce age-related protein aggregation, and promote cell/organismal robustness. While these programs appear to suppress the toxicity of disease associated proteins, they may also promote cancer.

HSF1 activity is controlled in cells through three broad mechanisms: (1) binding to molecular chaperones, such as HSP70, which prevents assembly of HSF1 into DNA binding competent trimers, (2) post-translational modification of HSF1 (phosphorylation, acetylation and SUMOylation), which can positively or negatively influence trimerization, nuclear localisation and DNA binding activity, and (3) opening or closing of chromatin at the promoters of HSF1 target genes (Gomez-Pastor et al., 2018; Vihervaara et al., 2018; Kmiecik and Mayer, 2022). Intriguingly, mitochondria appear to influence HSF1 activity by combining these mechanisms with reactive oxygen species (ROS) signaling to generate mitochondria-to-cytosolic stress responses that are distinct from that of the HSR (Wang and Chen, 2015; Wrobel et al., 2015; Kim et al., 2016; Williams et al., 2020; Aman et al., 2022) (Figure 1).

Increased levels of ROS (e.g., superoxide, hydrogen peroxide) are a common feature of mitochondrial dysfunction and have been proposed to act as important signalling molecules within cells (D'Autreaux and Toledano, 2007). Oxidation of yeast Ssa1(HSP70) at cysteines 264 and 303 by hydrogen peroxide, prevents its binding to, and repression of, HSF1. This leads to increased HSF1 activity and elevated levels of cytosolic HSPs. This was also observed with oxidomimetic Ssa1 mutants (Santiago and Morano, 2022). In addition, REDOX sensitive cysteine residues within the DNA binding domain of HSF1 itself, are also crucial for the upregulation of HSPs in response to heat or hydrogen peroxide treatment (Ahn and Thiele, 2003).

Similarly, studies in monkey kidney fibroblast-like (COS-7) cells, showed that treatment with the antioxidant N-acetyl-cysteine (NAC) suppressed activation of HSF1 in response to heat shock or treatment with the ROS generator, CCCP (an inhibitor of oxidative phosphorylation) (Agarwal and Ganesh, 2020). Activation of HSF1 by mitochondrial ROS was dependent on the perinuclear localisation of mitochondria, and was suppressed by treatment with the microtubule destabilising agent, nocodazole (Agarwal and Ganesh, 2020), suggesting that ROS from nuclear adjacent mitochondria may promote HSF1 activity in response to mitochondrial dysfunction.

ROS signaling has also been shown to be necessary, but not sufficient, for activation of the UPR^mt^ by HSF1 in HeLa cells. Mitochondrial ROS produced in response to mitochondrial protein misfolding leads to oxidation of cytosolic DNAJA1. This enhances the recruitment of HSP70 away from HSF1 and towards mitochondrial preproteins that accumulate within the cytosol. As a consequence, HSF1 is de-repressed and can drive the activation of the UPR^mt^, as well as a cytosolic stress response (Sutandy et al., 2023).

Together, these observations demonstrate that mitochondria use ROS signaling to communicate with HSF1 in order to drive both cytosolic and mitochondrial unfolded protein responses. Understanding exactly which forms of ROS impact HSF1 activity, and how this leads to transcriptional programs that are distinct from the HSR, may allow for the development of small molecules that promote HSF1 activity without the need for mitochondrial stress.

Intriguingly, a separate chaperone sequestration model has been proposed to underlie HSF1 activation upon loss of the mitochondrial outer membrane protein, MTCH-1. When MTCH-1 is depleted, HSP90 is titrated away from HSF-1 by inactive caspase-3, leading to the upregulation of cytosolic chaperones (Aman et al., 2022). This occurs without UPR^mt^ activation, suggesting that this results in a different form of HSF-1 than that which is responsive to ROS signaling. However, it remains unknown whether increased HSF-1 activity is due to the direct de-repression of HSF-1, increased misfolding of HSP90 clients, or both.

In addition to ROS signaling and chaperone sequestration, mitochondrial stress has also been reported to influence HSF1 activity through changes to its phosphorylation state. In response to heat shock, HSF1 becomes hyper-phosphorylated; in contrast, the activation of HSF-1 in response to ETC impairment in *C. elegans* is associated with dephosphorylation by the PP2A serine/threonine protein phosphatase complex (Williams et al., 2020). Intriguingly, activation of HSF1 by mitochondrial stress in mammalian cells is also independent of hyper-phosphorylation, while PP2A mediated dephosphorylation enhances HSF1 activity in cancer cells (Ishikawa et al., 2015; Asano et al., 2016; Katiyar et al., 2020).

The relationship between HSF1 activity and phosphorylation is highly complex (Budzynski et al., 2015); however, these reports suggest that changes in the phosphorylation status of HSF1 are required for the full activation of mitochondria-to-cytosolic stress responses. It is unclear if this is due to changes in interactions between HSF1 and specific repressors or activators, changes in DNA binding activity, or both. Given that PP2A activity is REDOX sensitive (Raman and Pervaiz, 2019), it will also be interesting to see what, if any, role ROS signaling has on influencing the phosphorylation status of HSF1. Furthermore, it remains unknown exactly which residues are dephosphorylated in response to mitochondrial stress and whether other PTMs and molecules beyond PP2A regulate HSF1 activity under conditions of mitochondrial dysfunction. For example, the metabolic stress sensors SIRT1 and AMPK are both activated in response to changes in mitochondrial homeostasis and have been shown to regulate HSF1 activity (Westerheide et al., 2009; Su et al., 2019).

As well as converging directly on the chemical landscape of HSF1 and its repressors, mitochondrial stress also promotes HSF1 activity through changes in chromatin architecture. In response to ETC impairment in worms, expression of the chromatin remodeler, ISW-1, is increased, which promotes the upregulation of sHSP coding genes by HSF-1 (Matilainen et al., 2017). In addition, the mitochondrial DNA replication factor, SSBP1, translocates to the nucleus in response to heat stress and potentiates HSF1 activity by recruiting the chromatin remodeling factor, BRG1, to HSF1 target promoters (Tan et al., 2015). These mechanisms promote cell survival and maintenance of mitochondrial homeostasis in response to proteotoxic stress (Tan et al., 2015; Matilainen et al., 2017). The mitochondrial protease, LONP1, has also been found to relocate to the nucleus and promote HSF1 activity in response to heat shock (Gibellini et al., 2022). However, the mechanism behind this remains to be elucidated.

Lastly, changes in the levels of specific lipids, particularly cardiolipin, has been found to be necessary for increased HSF-1 activity and activation of a mitochondria-to-cytosolic stress response in *C. elegans* following knockdown of mtHSP70 (Kim et al., 2016)*.* However, it remains unclear whether altered lipid homeostasis influences HSF-1 activity directly or indirectly.

### HSF1 preserves cell viability by increasing levels of cytosolic and mitochondrial chaperones

The upregulation of genes encoding sHSPs and HSP70 family members is commonly observed in response to mitochondrial stress, even though other cytosolic chaperones (e.g., HSP40, HSP90, TRiC) are not elevated. Why might this be beneficial to a full HSR?

sHSPs are ATP-independent molecular chaperones that assemble into a range of homo- and hetero-oligomeric structures that encase and sequester misfolded proteins. This allows cells to buffer against toxic preproteins in the cytosol without having to expend ATP that could be used elsewhere. The co-regulation of HSP70 in this response may allow cells to smoothly hand off proteins sequestered by sHSPs to HSP70 for refolding, trafficking to the proteasome, or perhaps even engagement with the mitochondrial import machinery once the stress is relieved. Another possibility is that elevated sHSPs also directly maintain protein homeostasis within mitochondria.

A recent study found that in multiple mammalian cells lines (HeLa, C2C12 and primary lymphoblasts) sHSPs are actively transported into mitochondria, where they form a competent proteostasis sub-network within the intermembrane space (IMS) (Adriaenssens et al., 2023). HSBP1, HSPB5 and HSPB8 all localised to the mitochondrial IMS, and this was strongly enriched following heat shock (Adriaenssens et al., 2023). Furthermore, simultaneous knockdown of sHSPs resulted in mitochondrial fragmentation and swelling, suggesting that increased HSF1 activity directly protects mitochondria through increasing levels of sHSPs.

Given that mitochondria-to-cytosolic stress responses are associated with many gene expression changes beyond the upregulation of molecular chaperones, it will be interesting to ascertain the full range of mechanisms by which HSF1 promotes mitochondrial homeostasis.

### Potential links between mitochondria-to-cytosolic stress responses and long-term tissue health

Consistent with its central role in safeguarding the proteome, HSF1 is essential for normal development, reproduction and lifespan in worms, flies, and mice (Jedlicka et al., 1997; Xiao et al., 1999; Hsu et al., 2003; Morley and Morimoto, 2004; Bierkamp et al., 2010; Li et al., 2016). HSF1 is also required for maintaining hematopoietic stem cells (HSCs), which is crucial for maintenance of the immune system and wound healing throughout life (Kruta et al., 2021). Development is associated with rapid cell growth and expansion of mitochondrial mass, and HSF-1 has been shown to regulate the expression of mitochondrial chaperones as part of a developmental program that is essential in *C. elegans* (Li et al., 2016). Therefore, it is possible that HSF1 promotes development, reproduction, and tissue homeostasis by supporting mitochondrial biogenesis early in life.

HSF-1 is also required for mild reductions in ETC activity to fully extend lifespan in worms (Labbadia et al., 2017), and over-expression or activation of HSF-1 has been reported to increase lifespan through elevated levels of histone H4, reduced expression of mtDNA encoded complex IV genes, and activation of the UPR^mt^ (Sural et al., 2020). Interestingly, the increased lifespan of worms overexpressing HSF-1 is also dependent on NHR-49 activity, the protection of actin networks and enhanced cytoskeletal integrity (Baird et al., 2014; Watterson et al., 2022), all of which have been shown to influence mitochondrial homeostasis (Pathare et al., 2012; Moore et al., 2016). These observations suggest that the pro-longevity effects of increased HSF-1 activity stem from the activation of pathways and mechanisms that converge on the regulation of mitochondrial networks.

In addition to positively influencing development and ageing, pharmacological and genetic activation of HSF1 has also been shown to suppress toxicity in worm, fly and mouse models of neurodegenerative disease (Huntington’s, Alzheimer’s, Parkinson’s and Amyotrophic Lateral Sclerosis) (Gomez-Pastor et al., 2018). In addition, increased levels of sHSPs have been proposed to suppress cardiovascular disease (Hu et al., 2017), while mutations in HSPB1 lead to Charcot-Marie-Tooth disease (HSPB1) (Szigeti and Lupski, 2009). Given that these diseases are also strongly associated with mitochondrial dysfunction, it is possible that activation of mitochondria-to-cytosolic stress responses might protect against disease progression by simultaneously providing support to distressed mitochondria while protecting the cytosol against the presence of toxic mutant proteins. In direct contradiction of this, a recent study has shown that mitochondria associated HSF1 exacerbates disease progression in cell, mouse and organoid models of Huntington’s disease (Liu et al., 2022). This is due to increased mitochondrial fragmentation and deletion of mtDNA (Liu et al., 2022).

While the positive effects of HSF1 on growth and longevity are generally beneficial, increased HSF1 activity is also linked to increased tumor incidence in mice and humans (Dai and Sampson, 2016). This is not associated with a HSR but is associated with changes in the expression of metabolic genes and HSP70 (Mendillo et al., 2012). Therefore, it is possible that HSF1 promotes cancer cell survival by promoting mitochondrial and metabolic remodeling, or that altered mitochondrial activity promotes HSF1 activity in cancer. This possibility is supported by the fact that ETC dysfunction leads to increased HSF1 activity in hepatomas, and that this is important for cell invasiveness (Lee et al., 2015). In addition, increased stiffness in the extracellular matrix (ECM) leads to a mechanosensitive signaling cascade that modulates mitochondrial network dynamics, increases ROS levels and activates an HSF1-dependent UPR^mt^ (Tharp et al., 2021). Many tumors exhibit altered ECM stiffness, therefore it is possible that this mechanism contributes to the activation of HSF1 in many cancers (Tharp et al., 2021).

## Conclusion

It has become clear that HSF1 promotes mitochondrial homeostasis through the activation of mitochondrial-to-cytosolic stress responses and regulation of the UPR^mt^. While the mechanisms behind this are still emerging, it remains unclear exactly how mitochondria generate a form of HSF1 that drives a transcriptional program that is distinct from the HSR. Is this due to changes in the DNA binding activity of HSF1, changes in promoter accessibility, or perhaps more likely, interactions with as yet unidentified mitochondrial stress specific co-activators. In addition, it is unclear why different types of mitochondria-to-cytosolic stress response exist, and whether these are active in all tissues throughout life. One plausible scenario is that the existence of different responses reflects accompanying variations in metabolism and the proteins that are most at risk of misfolding. Addressing these questions may help us to develop specific activators and inhibitors of mitochondria-to-cytosolic stress responses that can be used to promote healthy human ageing.
